# Supplementary material for: Analysis of chi angle distributions in free amino acids via multiplet fitting of proton scalar couplings
Source: Magn Reson (Gott). 2024 Aug 19;5(2):103–20. doi: 10.5194/mr-5-103-2024 (PMC11570886; doi:10.5194/mr-5-103-2024)
Supplement: The supplement related to this article is available online at: https://doi.org/10.5194/mr-5-103-2024-supplement. [file mr-5-103-2024-supplement.zip › Syed2024/data/fit1d_fitnmr_output.tar.gz!/fit1d/lys/500/bmse000043_simulation_1_nmredata/1H/resonances_1d.pdf]

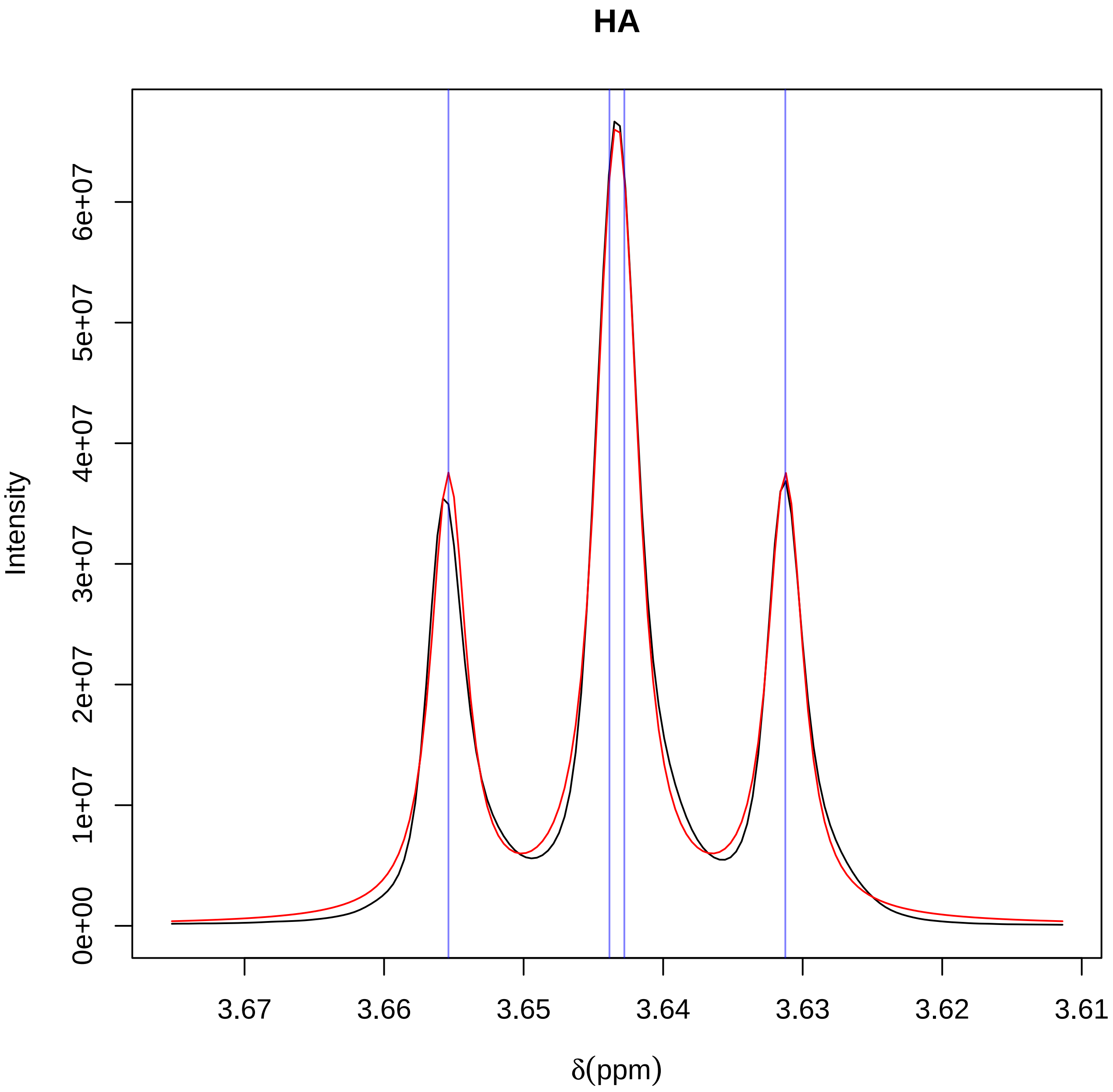

HA
Intensity
0e+00
1e+07
2e+07
3e+07
4e+07
5e+07
6e+07
3.67
3.66
3.65
3.64
3.63
3.62
3.61
δ(ppm)

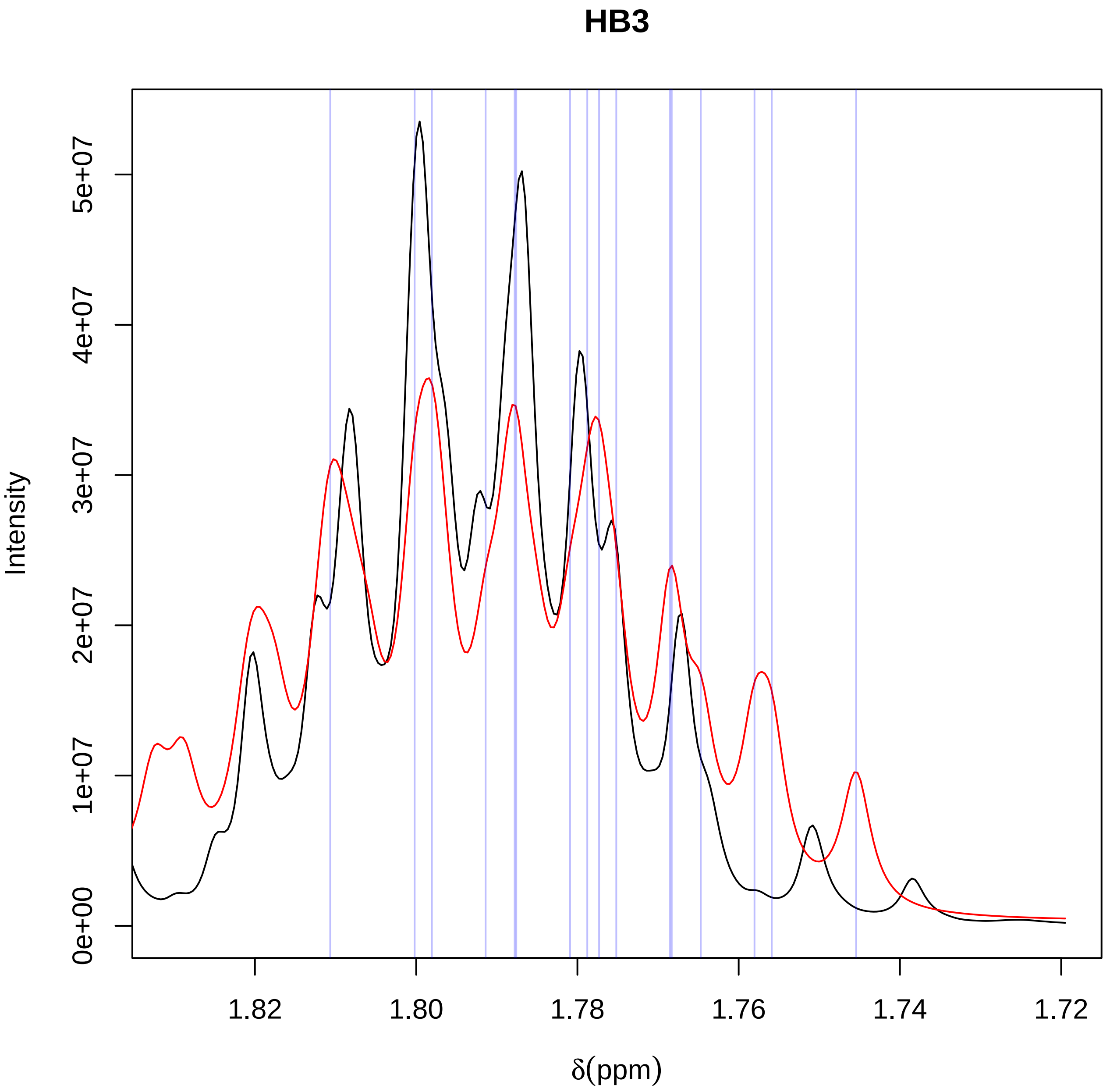

HB3
Intensity
0e+00
1e+07
2e+07
3e+07
4e+07
5e+07
1.82
1.80
1.78
1.76
1.74
1.72
δ(ppm)

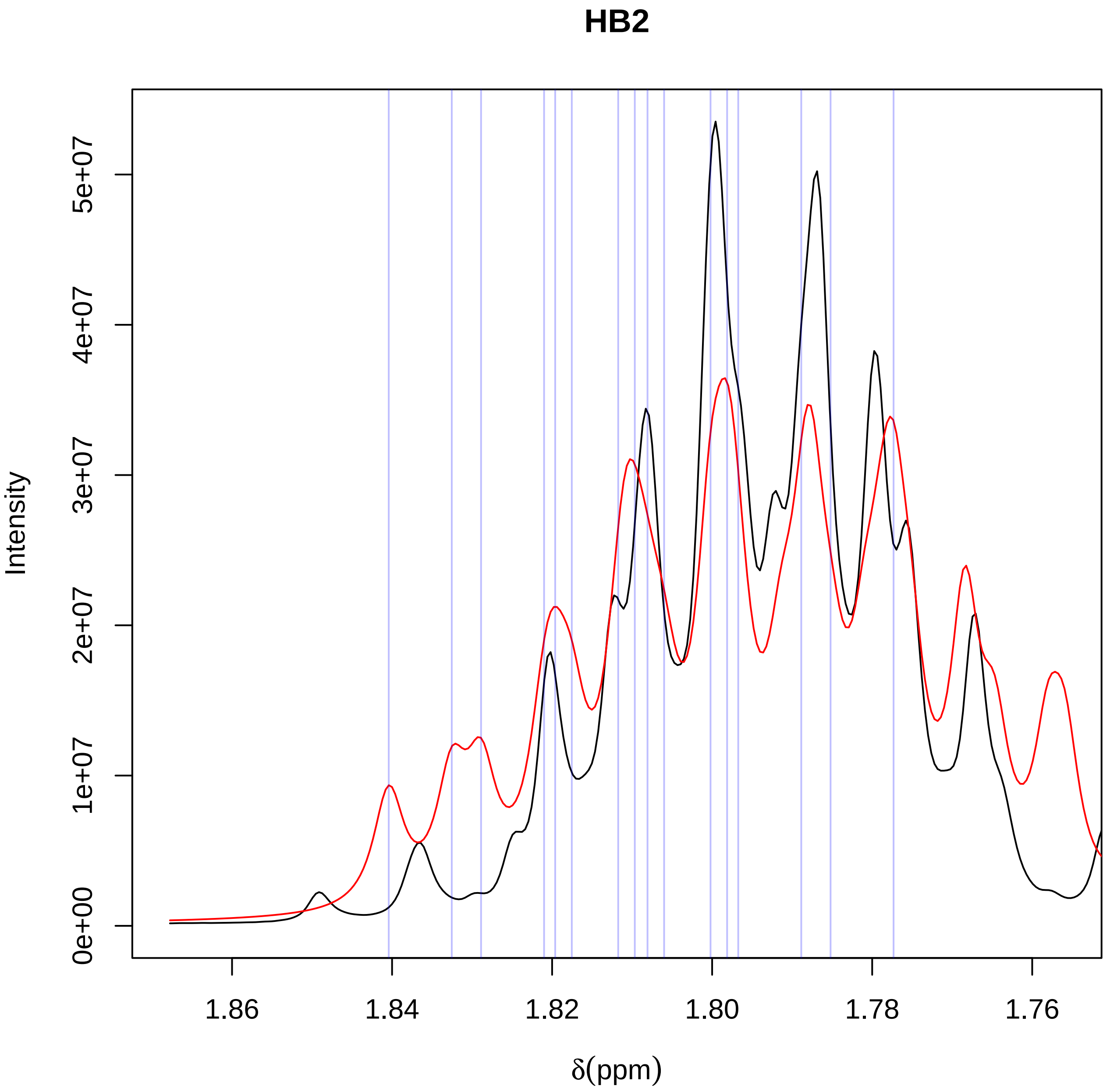

HB2
Intensity
δ(ppm)
0e+00
1e+07
2e+07
3e+07
4e+07
5e+07
1.86
1.84
1.82
1.80
1.78
1.76

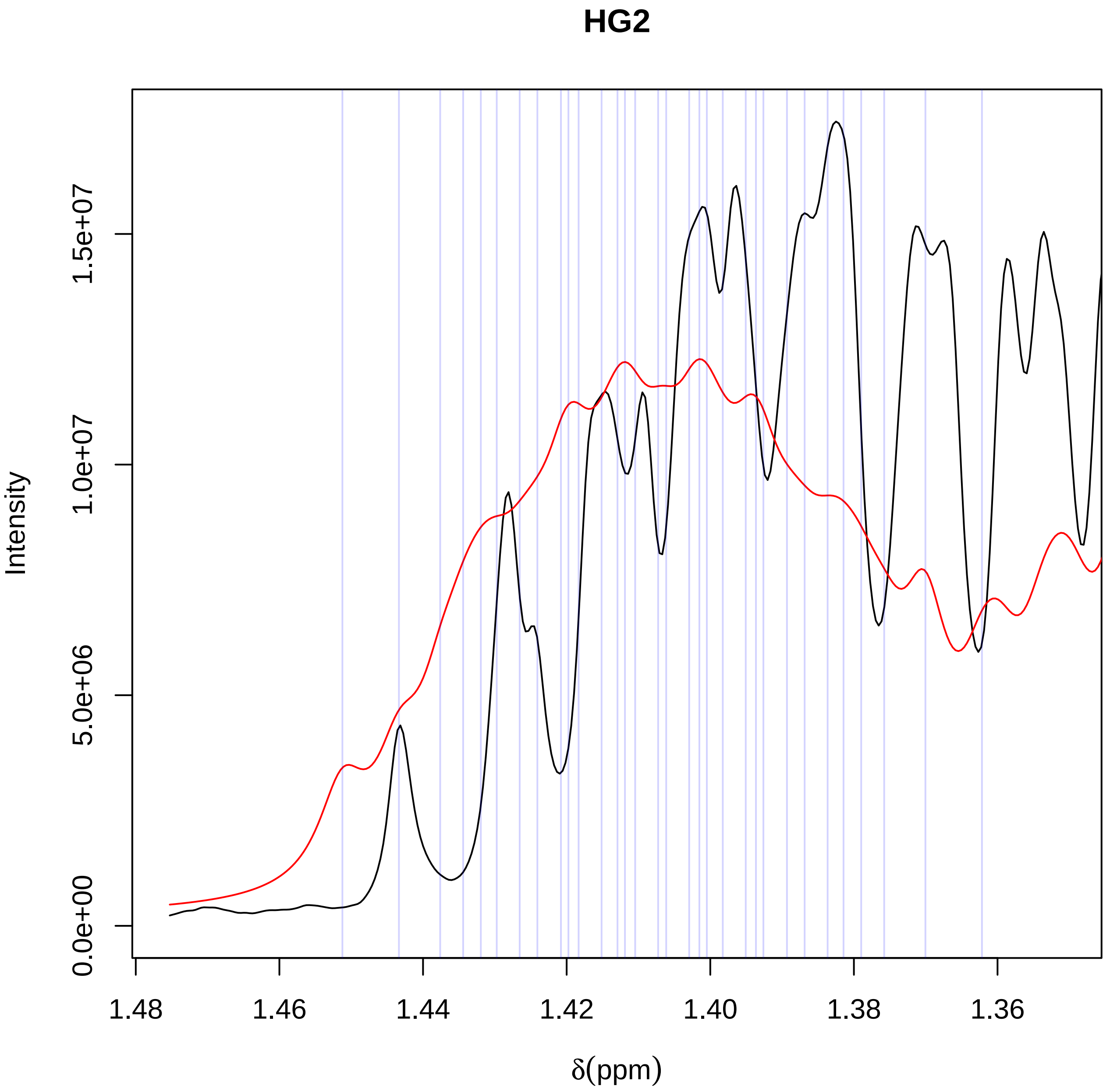
HG2
Intensity
0.0e+00
5.0e+06
1.0e+07
1.5e+07
1.48
1.46
1.44
1.42
1.40
1.38
1.36
δ(ppm)

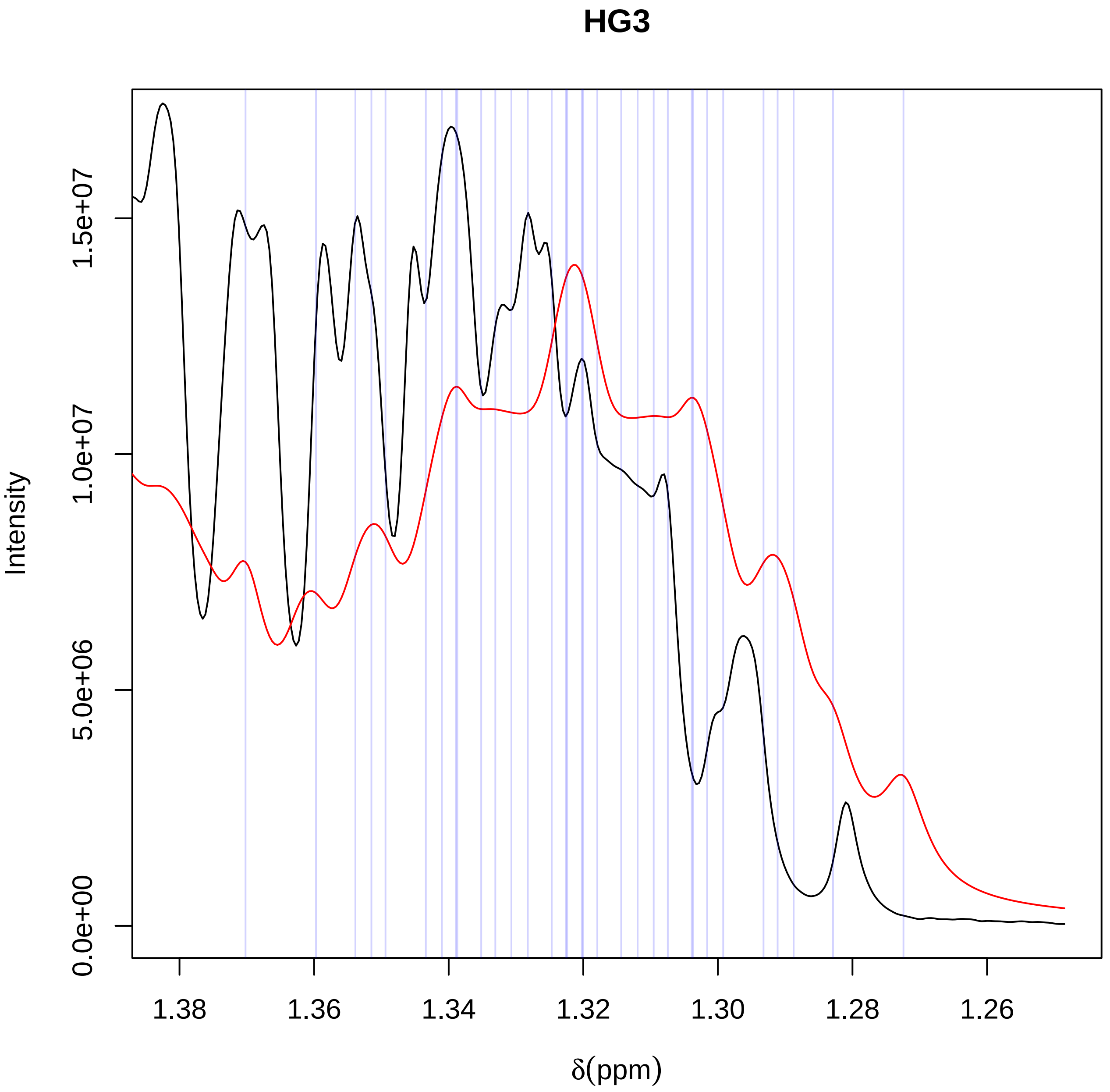

HG3
Intensity
0.0e+00
5.0e+06
1.0e+07
1.5e+07
1.38
1.36
1.34
1.32
1.30
1.28
1.26
δ(ppm)

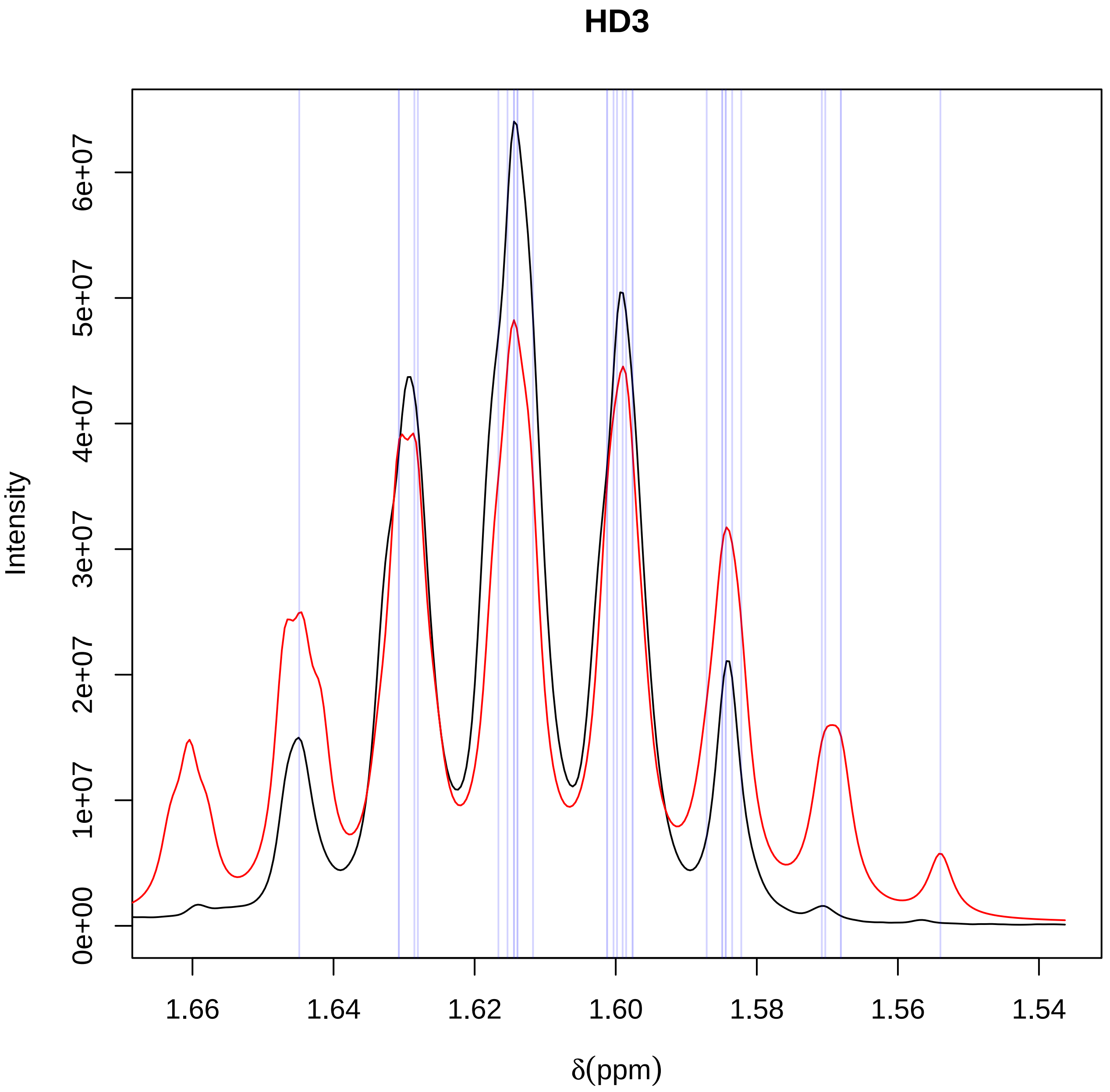

HD3
Intensity
0e+00
1e+07
2e+07
3e+07
4e+07
5e+07
6e+07
1.66
1.64
1.62
1.60
1.58
1.56
1.54
δ(ppm)

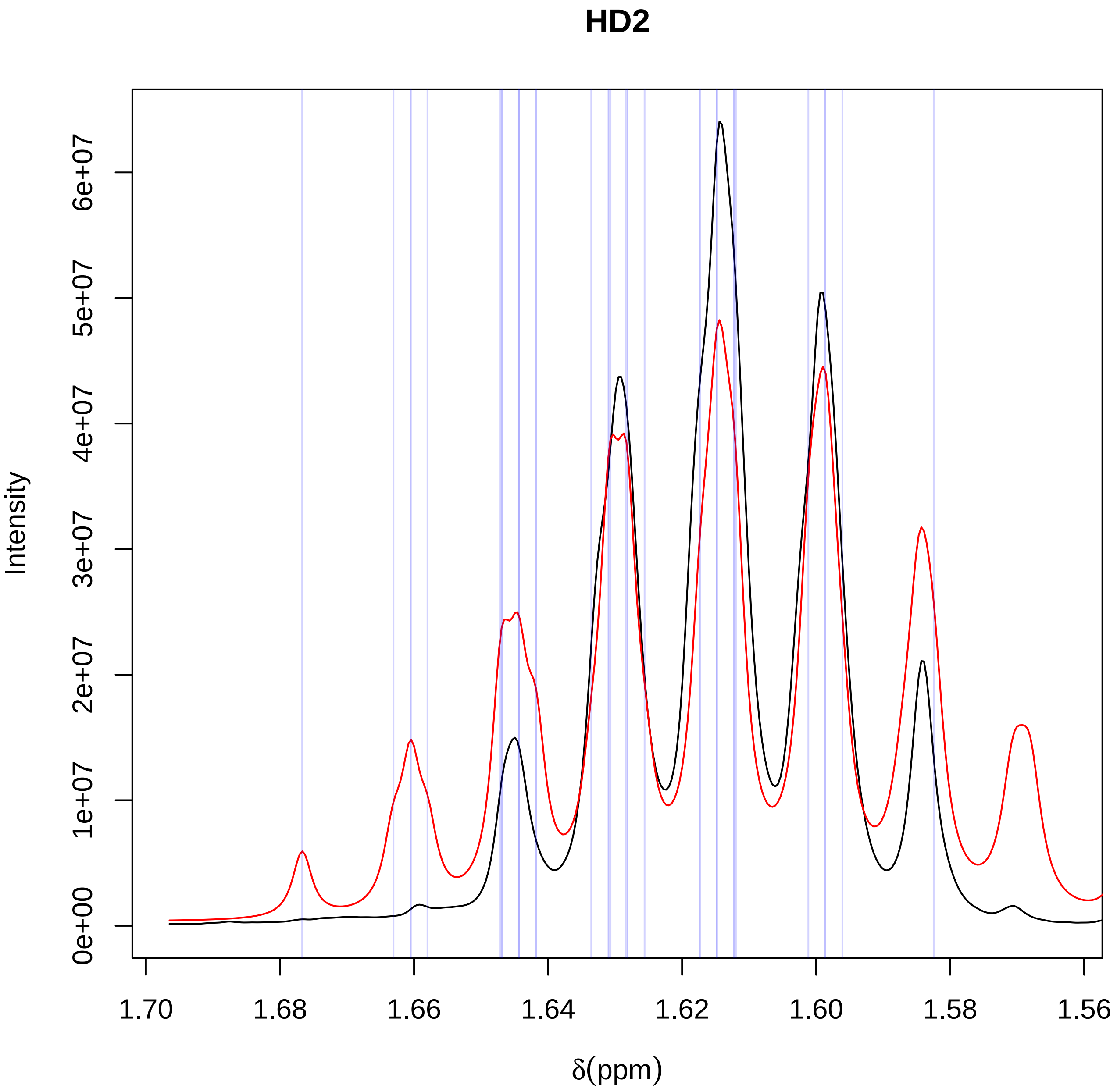
HD2
Intensity
0e+00
1e+07
2e+07
3e+07
4e+07
5e+07
6e+07
1.70
1.68
1.66
1.64
1.62
1.60
1.58
1.56
δ(ppm)

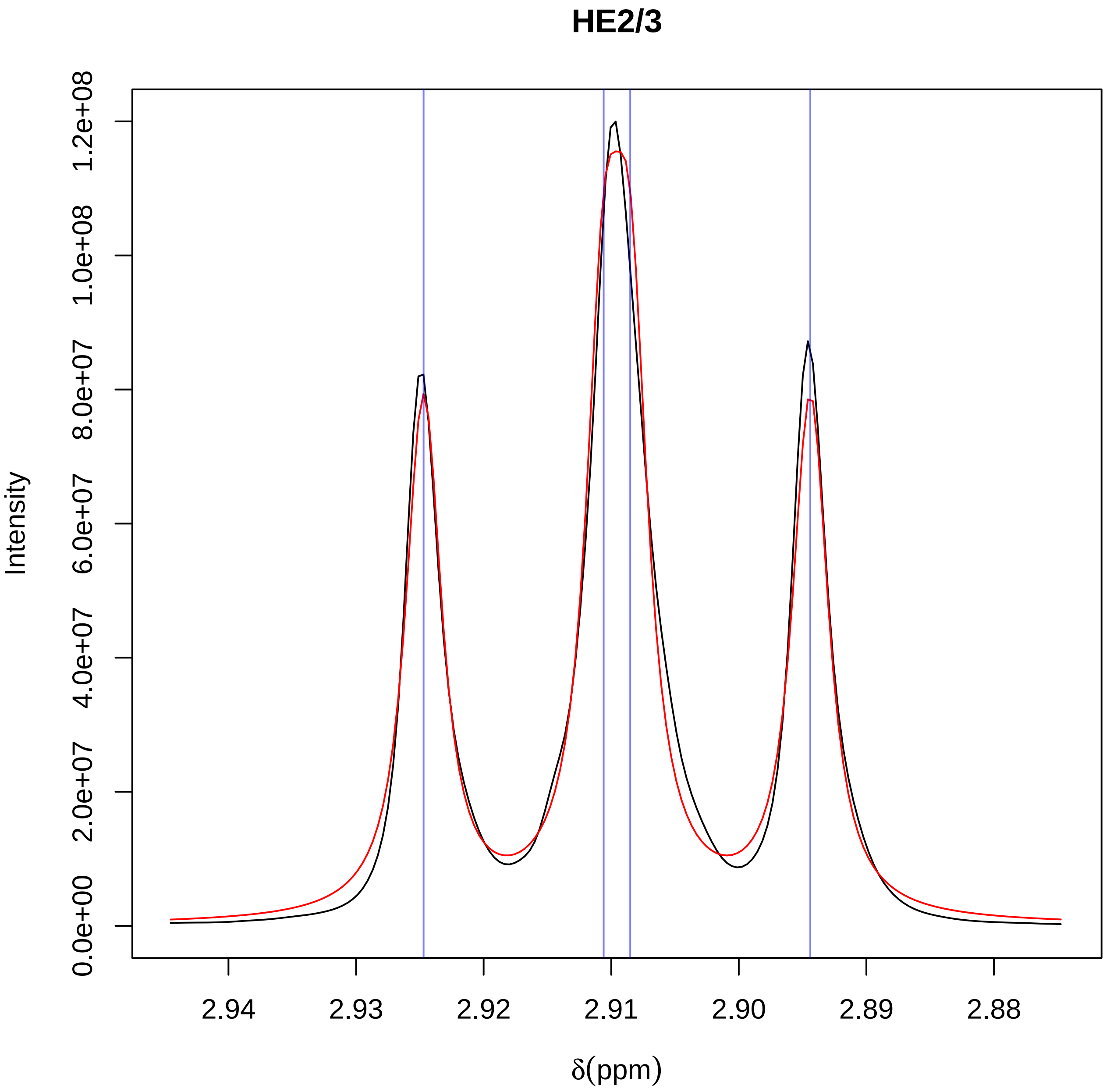

HE2/3
Intensity
0.0e+00
2.0e+07
4.0e+07
6.0e+07
8.0e+07
1.0e+08
1.2e+08
2.94
2.93
2.92
2.91
2.90
2.89
2.88
δ(ppm)
